# Non-canonical functions of EZH2 in cancer

**DOI:** 10.3389/fonc.2023.1233953

**Published:** 2023-08-18

**Authors:** Sarah M. Zimmerman, Phyo Nay Lin, George P. Souroullas

**Affiliations:** ^1^ Department of Medicine, Washington University School of Medicine in St. Louis, St. Louis, MO, United States; ^2^ Division of Oncology, Molecular Oncology Section, Washington University School of Medicine in St. Louis, St. Louis, MO, United States; ^3^ Siteman Comprehensive Cancer Center, Washington University School of Medicine in St. Louis, St. Louis, MO, United States

**Keywords:** EZH2, H3K27me3, non-canonical, cancer, oncogene, tumor suppressor

## Abstract

Mutations in chromatin modifying genes frequently occur in many kinds of cancer. Most mechanistic studies focus on their canonical functions, while therapeutic approaches target their enzymatic activity. Recent studies, however, demonstrate that non-canonical functions of chromatin modifiers may be equally important and therapeutically actionable in different types of cancer. One epigenetic regulator that demonstrates such a dual role in cancer is the histone methyltransferase EZH2. EZH2 is a core component of the polycomb repressive complex 2 (PRC2), which plays a crucial role in cell identity, differentiation, proliferation, stemness and plasticity. While much of the regulatory functions and oncogenic activity of EZH2 have been attributed to its canonical, enzymatic activity of methylating lysine 27 on histone 3 (H3K27me3), a repressive chromatin mark, recent studies suggest that non-canonical functions that are independent of H3K27me3 also contribute towards the oncogenic activity of EZH2. Contrary to PRC2’s canonical repressive activity, mediated by H3K27me3, outside of the complex EZH2 can directly interact with transcription factors and oncogenes to activate gene expression. A more focused investigation into these non-canonical interactions of EZH2 and other epigenetic/chromatin regulators may uncover new and more effective therapeutic strategies. Here, we summarize major findings on the non-canonical functions of EZH2 and how they are related to different aspects of carcinogenesis.

## Introduction

**Figure 1:**
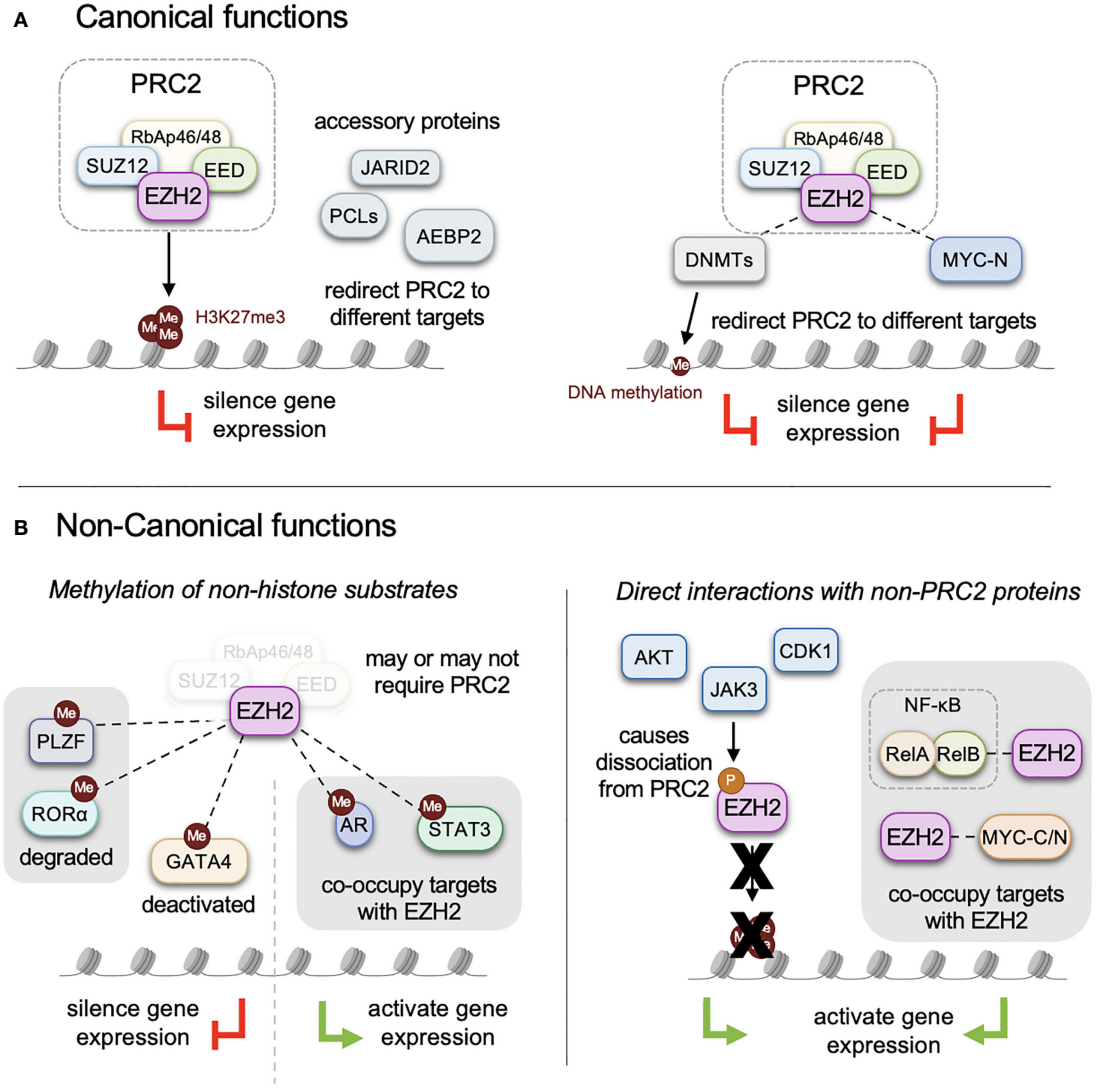
Canonical and non-canonical functions of EZH2. **(A)** EZH2 functions as the methyltransferase domain of the PRC2 complex in cooperation with core components, EED, SUZ12 and RbAp46/48, to mediate methylation of lysine 27 on histone 3 (H3K27me3). Several accessory proteins that enhance and direct PRC2 function include JARID2, PCLs, AEBP2 among others. **(B)** In addition to methylating histone tails EZH2 can also methylate non-histone proteins, altering their function and activity with complicated effects on downstream gene expression. These include PLZF, RORα, GATA4, AR and STAT3. Post-translational modifications of EZH2 by AKT, JAK3, CDK1, and protein interactions with non-PRC2 proteins promote non-canonical functions, which can inhibit canonical PRC2 assembly and histone methylation. PRC2-independent functions of EZH2 include interactions with transcription factors such as RelA/B, CMYC, NMYC, STAT3, where they together function as transcriptional activators.

Polycomb group proteins were originally described for their role in *Drosophila* development where they repress homeotic genes (Hox) required for proper body segmentation (1–3). The two main Polycomb group complexes are the Polycomb repressive complex 1 (PRC1) and Polycomb repressive complex 2 (PRC2), both of which are conserved from *Drosophila* to mammals. While PRC1 consists of highly variable subunits, PRC2 is composed of four core components: EZH1 or EZH2, EED, SUZ12 and RbAp46/48. Additionally, several secondary accessory proteins such as JARID2, PCL and AEBP2 modulate the function of PRC2 (4) (**Figure 1A**). Polycomb-mediated gene silencing primarily operates through the regulation of chromatin structure via post-translational modifications of histone tails (5, 6). Due to their involvement in critical biological processes like differentiation, cell identity, proliferation, stemness, and plasticity, alterations in Polycomb complexes are frequently associated with human cancers (7, 8) and developmental syndromes characterized by congenital overgrowth, dysmorphic facial features, and learning disabilities (9–13).

In the context of cancer, the role of PRC2, and EZH2 specifically, is multifaceted as it can function both as a tumor-suppressor (14–18) and as an oncogene (17, 19–23) in a cell type dependent manner. This dual functionality is reflected in the observation of EZH2 amplifications and deletions in different kinds of cancer, which have been validated through functional assays and *in vivo* models. In addition to loss- and gain-of-function genetic events, missense mutations at tyrosine 641 (EZH2^Y641^) function in a neomorphic manner, cause redistribution of H3K27me3 and are strongly oncogenic (19).

While much of the oncogenic activity of PRC2, and particularly EZH2, has been attributed to its enzymatic activity of methylating lysine 27 on histone 3, recent studies revealed that this canonical enzymatic activity cannot fully explain all of its physiological functions and oncogenic effects. Considering the limited efficacy of epigenetic inhibitors in cancer patients, which primarily target enzymatic activity, a more focused investigation into EZH2’s non-canonical functions may identify more effective therapeutic strategies. In this review, we highlight and discuss the major non-canonical functions of EZH2, its interactions with non-PRC2 proteins, along with implications on therapeutic strategies.

## Post-translational modifications of EZH2 may determine non-canonical functions

Several post-translational modifications of EZH2 have been identified over the years which may be responsible for switching between canonical and non-canonical EZH2 functions (**Figure 1B**). A study by Cha et al. was the first to find that the AKT kinase phosphorylates EZH2 at serine 21 (S21), resulting in decreased methyltransferase activity (24). Later studies found that phosphorylation at S21 not only suppressed canonical enzymatic activity, but was required for non-canonical, H3K27me3-independent functions of EZH2 in prostate cancer (25) and glioblastoma models (26). Other kinases known to modify EZH2 include JAK3 and the cyclin dependent kinases 1 and 2 (CDK1/2). JAK3 phosphorylates EZH2 at tyrosine 244, promoting the dissociation of the PRC2 complex (27), while CDKs have a more complicated effect; CDK1/2 phosphorylate EZH2 at threonine 350, enhancing recruitment of PRC2 and associations with non-coding RNAs (28), while CDK1-mediated phosphorylation of EZH2 at threonine 487 disrupts binding with PRC2 core components EED and SUZ12, inhibiting H3K27me3 (29). Different modifications, therefore, could have very different effects on the ability of EZH2 to form protein complexes with either canonical PRC2 components, or other effectors, which may be dependent on the right conformational structure. While many of these modifications are known and contribute or promote non-canonical EZH2 functions as discussed further below, many likely remain unexplored.

## Direct interaction between Ezh2 and DNA methyltransferases in solid tumors

One of the earliest interaction of EZH2 outside of core PRC2 components and accessory proteins was with the DNA methyltransferases, DNMT1, 3A and 3B (30). Vire et al. found that EZH2 directly interacts and recruits DNMTs to specific target loci (**Figure 1A**). Repression of these genes required the presence of both H3K27me3 and DNA methylation in the promoter region. Cooperation between these two epigenetic complexes also had significant therapeutic implications with combination strategies targeting both DNMT and EZH2 activity. For example, Ning et al. showed that in gastric cancer and glioblastoma cell lines EZH2 and DNMT1 together repressed expression of tumor suppressors and that treatment with either the DNMT inhibitor 5-azadeoxycitidine and the methyltransferase inhibitor DZNep reduced tumor growth (31). These early studies, however, were limited by the fact that DZNep is not a specific EZH2 inhibitor and enhanced efficacy could be due to inhibition of other methyltransferases. Later studies with more specific EZH2 inhibitors showed that in hepatocellular carcinoma the combination of 5-azadeoxycitidine with the EZH2 inhibitor GSK126 increased expression of neoantigens, increased tumor-infiltrating lymphocytes and reduced cell growth (32, 33). In multiple myeloma, Dimopoulos et al. investigated resistance to lenalidomide and pomalidomide and found that resistance was associated with increased DNA methylation and decreased chromatin accessibility (34). Combination treatment with 5-azacytidine and the EZH2 inhibitor, EPZ-6438, reversed these epigenetic changes and re-sensitized the cells to lenalidomide and pomalidomide. Dual inhibition of DNMTs and EZH2 was also effective in prostate, breast, colon cancer and leukemia cells (35, 36), highlighting the therapeutic potential of these interactions.

## Ezh2-mediated methylation of Gata4 and its role in rhabdomyosarcoma

In addition to its interactions with other epigenetic regulators, Ezh2 can directly interact with transcription factors, such as Gata4 (**Figure 1B**). Gata4 is a key transcription factor during heart development that recruits the acetyltransferase p300 to specific chromatin loci, activating the expression of genes critical to heart development (37). While p300 acetylates Gata4 enhancing its transcriptional activity (38), Ezh2 antagonizes this interaction by directly methylating Gata4 on lysine 299 (39). This prevents its acetylation and activation by p300, reducing its recruitment to chromatin and impeding the activation of the heart development program. This was the first report of a non-histone interaction of EZH2 and an example of a histone methyltransferase and a histone acetyltransferase that function antagonistically in a histone-independent manner. A direct interaction between EZH2 and GATA4 was also observed in rhabdomyosarcoma, a type of soft tissue sarcoma that develops from muscle or fibrous tissue, and a rare type of sarcomas of the heart. Song et al. demonstrated that GATA4 promotes normal myogenesis by binding to GRIP1 and stimulating the expression of miR-29a, which drives myogenic differentiation (40). However, in sarcoma cells, a direct interaction between GATA4 and EZH2 leads to the deposition of H3K27me3 at the miR-29a promoter suppressing its expression, inhibiting myogenic differentiation while promoting proliferation. Overall, these results suggest that interactions of GATA4 with PRC2 components are important in rhabdomyosarcoma progression.

## Transcriptional activation mediated by EZH2 and the androgen receptor in prostate cancer

While many EZH2 interactions require the presence of the rest of the PRC2 complex as described above, EZH2 can engage in protein-protein interactions independently of other PRC2 components or activate downstream signaling independent of its methyltransferase activity. An early example where EZH2 functions as an activator is in androgen-dependent prostate cancer (23). Varambaly et al. initially found that EZH2 expression was highly correlative with progression of castration-resistant prostate cancer (23). Subsequent studies found that silencing of EZH2 had a profound effect on androgen-independent cell growth *in vitro*, with a significant number of genes directly upregulated by EZH2 (25). ChIP-seq experiments for EZH2 and H3K27me3 identified both EZH2-specific peaks, termed “solo”, as well as peaks overlapping with H3K27me3, termed “ensemble”. Additional mechanistic studies found that EZH2 directly methylated the Androgen receptor (AR), and together with AR, regulated expression of activated genes. Xu et al. further suggested that the EZH2-AR interaction was dependent on phosphorylation of EZH2 at serine 21. Interestingly, this interaction still required the intact methyltransferase activity of EZH2, although the mechanistic details remain unclear. In contrast to Xu et al., Kim et al. proposed that EZH2 directly binds to the promoter of the AR, controlling its expression independent of its methyltransferase activity (41). Differences in methodology and models could account for these disparate results and both mechanisms could be true.

## Non-canonical functions of EZH2 in breast cancer involve transcriptional activation and protein degradation mechanisms

In breast cancer, EZH2 plays a significant role via both canonical and non-canonical functions, which are summarized by Anwar et al. (42). Interestingly, the non-canonical function of EZH2 in breast cancer is not the same across different subtypes. In triple-negative breast cancer, EZH2 acts as a transcriptional activator of the NF-KB subunit RelB to drive self-renewal and promote tumor initiation (43, 44). In Estrogen Receptor (ER)-negative breast cancer, EZH2 physically interacts with RelA/RelB and promotes the transcription of NF-KB targets such as IL6 and TNF, independent of its histone methyltransferase activity. However, in ER-positive breast cancer EZH2 assumes its canonical role, interacting with the ER in a PRC2-dependent manner mediating H3K27me3 at NF-KB target genes and repressing their expression (44).

EZH2 also non-canonically regulates gene expression by targeting proteins for degradation via E3 ubiquitin ligases. Lee et al. discovered that in breast cancer cells EZH2 recognizes and monomethylates the lysine of an Arginine(R)-Serine(S)-Lysine(K) histone-like sequence on RORα, leading to its subsequent ubiquitination and degradation by DCAF1 (45). These data may be biologically significant in cancer as well, as expression of RORα and EZH2 is inversely correlated in breast cancer tissues (45).

## Ezh2 association with protein degradation machinery in nature killer T cell lymphoma

Interactions of EZH2 with protein degradation machinery are not unique to breast cancer. While studying the role of EZH2 during T cell development, Vasanthakumar et al. found diametrically different results when deleting different core components of the PRC2 complex; depletion of Ezh2 resulted in expansion of NKT cells, while depletion of Eed and Suz12 resulted in a dramatic loss of NKT cells (46). They subsequently found that PLZF, a master regulator of hematopoiesis and NKT cell expansion (47) was directly methylated by EZH2 at lysine 430, which lead to its ubiquitination and degradation, providing an explanation for the observed phenotypes. EZH2 and PLZF also interact directly on chromatin in myeloblastic cells, co-occupying loci that are not associated with SUZ12, EED, or H3K27me3, but are instead associated with H3K4me3 and active transcription (48), highlighting the multilevel complexity of EZH2’s functions.

## Cell-intrinsic and cell-extrinsic effects of an EZH2-STAT3 interaction in multiple solid tumors

A direct interaction between EZH2 and the immune-regulator, STAT3, has been observed in multiple solid tumors. In melanoma, EZH2 is either amplified, overexpressed or mutated at Tyrosine 641 (Y641), a hotspot mutation within the methyltransferase domain which creates a hyperactive and neomorphic protein (19). To understand the mechanisms of the oncogenic activity of this mutant, we recently investigated potential non-canonical interactions in melanoma and found that in mouse melanoma cells, mutant Ezh2^Y641F^ protein interacts directly with and methylates Stat3 protein (49). We found that the interaction between Ezh2 and Stat3 was highly enriched in the presence of mutant *Ezh2* ^Y641F^ protein, which exhibits increased methyltransferase activity, suggesting that this interaction is dependent on Ezh2’s activity. Using chromatin-immunoprecipitation followed by sequencing (ChIP-seq) and expression profile analysis, we found that Ezh2^Y641F^ and Stat3 cooperate as transcriptional activators, binding to new regions on chromatin to increase expression of several target genes. These include MHC Class 1b antigen processing genes and autophagy regulators. Functionally, the Ezh2^Y641F^-Stat3 interaction mediated the recruitment of cytotoxic CD8+ T cells to the melanoma tumor microenvironment, which slowed down *in vivo* tumor growth (49). This was the first report of an enhanced non-canonical interaction between the Ezh2^Y641F^ mutant and a non-histone protein, suggesting that perhaps this is an important aspect of the oncogenic activity of EZH2 in cancers with Y641 mutations (7, 8).

The interaction between EZH2 and STAT3 has also been reported in glioblastoma and colon carcinomas. In glioblastoma, EZH2 binds to and methylates STAT3 at lysine 180, resulting in enhanced STAT3 activity (26), while in colon carcinoma Dasgupta et al. found that dimethylation of STAT3 at lysine 49 was crucial for activating the IL6-STAT3 transcriptional program (50). EZH2 can also methylate STAT3 in breast cancer cells, which was necessary for tumor growth (51). Overall, the function and downstream mechanisms of the EZH2-STAT3 interaction appears to be determined by cellular context and may depend on availability of other co-factors or post-translational modifications.

## Multifaceted interactions between EZH2 and MYC in both solid and blood cancers

MYC is a well-studied oncogene that is implicated in many types of cancers. The relationship between MYC and EZH2 was established more than a decade ago with studies demonstrating their co-regulation. For example, EZH2 directly regulates CMYC expression via association with the estrogen receptor alpha (ERα) and β-catenin (52), and conversely, MYC binds the promoter of EZH2 and regulates its expression in prostate cancer cells (53) and B cell lymphomas (19). The latter raised the intriguing possibility that EZH2 may be mediating some of the oncogenic activity of MYC and account for the repressed genes in cancers with MYC overexpression. This was demonstrated by Kaur and Cole in immortalized mammary epithelial cells. Specifically, they showed that MYC transcriptionally activates PTEN, leading to suppression of AKT kinase activity, which in turn results in increased EZH2 protein stability and enzymatic activity. They further showed with rescue assays and EZH2 mutants that EZH2 was responsible for the transcriptional repression of nearly half of all the MYC-repressed genes, consistent with EZH2 binding and accumulation of H3K27me3 at MYC-repressed gene loci (54).

While the above studies suggested EZH2/PRC2 may be mediating some of the repressive properties of MYC through indirect signaling, they did not exclude the possibility of a more direct interaction. More recent studies proposed that possibility, but it remained unclear how EZH2 and MYC interacted at the chromatin interface. Two recent studies shed more light into these questions by investigating the interaction between EZH2 and CMYC in acute leukemia and multiple myeloma models (55, 56). Specifically, Wang J et al. discovered that EZH2 not only binds to regions characterized by repressive H3K27me3 marks, as is typical of canonical EZH2 activity, but also to regions with no H3K27me3 (termed “EZH2-solo” sites). In fact, significant EZH2 binding overlaps with multiple gene-active chromatin marks, such as H3K4me3, H3K9ac and H3K27ac. Interestingly, they found that these EZH2-solo sites were also bound by CMYC. They further demonstrated that EZH2 and CMYC directly interact through EZH2’s transactivation domain (TAD) and that this interaction is essential for malignant growth of leukemia cells. This result was consistent with a previous report that identified a hidden TAD within EZH2 that can be unlocked by cancer-specific EZH2-phosphorylation, resulting in structural conformation changes that mediate interactions with active transcription machinery (57).

In addition to interacting with CMYC, EZH2 also interacts with NMYC, both in a canonical and non-canonical way. In neuroblastoma cells, Corvetta et al. demonstrated that NMYC directly interacts with EZH2 and recruits the PRC2 complex to the promoter region of the tumor suppressor, clusterin (CLU). This resulted in increased deposition of H3K27me3, suppressing expression of CLU, and causing significant phenotypic effects such as increased invasion and cell cycle progression (58). In prostate cancer cells, Dardenne et al. showed that NMYC forms a complex with EZH2 and the Androgen Receptor (AR), which enhances PRC2 target gene repression, regulating gene expression programs critical for prostate cancer progression (59). Contrary to these canonical functions of EZH2 in cooperation with NMYC, Vanden Bempt et al. recently found that EZH2 exhibits non-canonical functions in coordination with NMYC in peripheral T-cell lymphoma (PTCL). Specifically, using a mouse model of NMYC-driven PTCL, they found that EZH2 acts as a cofactor of NMYC, activating expression of EZH2-NMYC bound sites without association with other canonical PRC2 components or activity (60).

A noteworthy observation from these studies was that the interaction between EZH2 and MYC was unique to transformed leukemia cell lines and primary leukemia patient samples, and was not detected in healthy, non-leukemic cells. This suggests that the EZH2-MYC interactions may depend on the presence of specific co-factors or post-translational modifications that facilitate that interaction and are not present in healthy cells or in all types of cancers.

## Therapeutic implications of EZH2 non-canonical functions

To investigate the importance of EZH2’s canonical and non-canonical activities in cancer and therapeutics, several studies have explored the concept of synthetic lethality between EZH2 inhibition and mutations in other pathways. A notable example is the relationship between PRC2/EZH2 and the SWI/SNF complex in cancer. Several cancers with mutations in SWI/SNF subunits exhibit increased sensitivity to PRC2 inhibition (61, 62). However, Kim et al. demonstrated that SWI/SNF-mutant lung and ovarian cancer cells are primarily dependent on the non-catalytic functions of EZH2 and only partially dependent on its methyltransferase activity (61). This strongly suggests that non-enzymatic functions of EZH2 are not only relevant in cancer progression, but also to therapeutic approaches.

The non-canonical functions of EZH2 may also explain the relatively weak responses of EZH2 inhibitors in some clinical trials and must be taken into serious consideration when designing therapeutic strategies. Several recent studies have utilized proteolysis targeting chimeras (PROTACs) technology to hijack the ubiquitin-proteasome pathway to degrade targeted proteins for loss-of-function studies. Degradation of EZH2 by the PROTAC-degrader MS-177 effectively inhibited on-target EZH2-PRC2 and MYC-related oncogenic nodes and inhibited leukemia growth *in vivo* more effectively than enzymatic inhibition (55, 56). In a separate study, using the EZH2 PROTAC-degrader MS1973, EZH2 degradation was necessary and more effective than enzymatic inhibition in eliminating relapse-initiating cells in a retinoic acid-dependent model of acute promyelocytic leukemia (63). These studies highlight the potential clinical significance of targeting non-canonical functions of EZH2, and may even be more broadly applicable to other chromatin-modifying genes.

## Discussion

The non-canonical interactions of EZH2 encompass a wide range of functions. Recent studies provided significant insights into the intricate roles of epigenetic regulators and their interactions with oncogenes such as MYC. It is important to acknowledge that while these interactions are intriguing, many of them have yet to be validated *in vivo*. Considering that the proper biological context greatly influences protein functionality, it is possible that these interactions may be different *in vivo*. Nonetheless, these studies underscore the notion that proteins often have additional roles beyond their established functions as epigenetic regulators or chromatin modifiers. These additional roles can arise from association with other protein complexes aberrantly expressed in cancer cells, or by post-translational modifications, which can cause structural conformational changes, facilitating novel interactions not typically present in healthy cells. Considering these discoveries, targeting the degradation of EZH2, or its post-translational modifiers could be a promising therapeutic approach for cancers that rely on these non-canonical interactions. However, the specificities of these interactions to cancer cells, their occurrence in different contexts, and the involvement of other epigenetic regulators in similar functionalities are still not fully understood. Ongoing and future research will undoubtedly uncover more of these interactions, providing a deeper understanding of disease mechanisms and guiding the development of effective therapeutic strategies.

## Author contributions

All authors researched the literature and wrote the manuscript with GPS’s supervision. All authors contributed to the article and approved the submitted version.
